# A Plant Extract of *Ribes nigrum folium* Possesses Anti-Influenza Virus Activity *In Vitro* and *In Vivo* by Preventing Virus Entry to Host Cells

**DOI:** 10.1371/journal.pone.0063657

**Published:** 2013-05-23

**Authors:** Christina Ehrhardt, Sabine Eva Dudek, Magdalena Holzberg, Sabine Urban, Eike Roman Hrincius, Emanuel Haasbach, Roman Seyer, Julia Lapuse, Oliver Planz, Stephan Ludwig

**Affiliations:** 1 Institute of Molecular Virology (IMV), Centre of Molecular Biology of Inflammation (ZMBE), Westfälische Wilhelms-University of Muenster, Muenster, Germany; 2 Interfaculty Institute for Cell Biology, Department of Immunology, University of Tuebingen, Tuebingen, Germany; 3 Dr. Pandalis NatUrprodukte GmbH, Glandorf, Germany; Lady Davis Institute for Medical Research, Canada

## Abstract

Infections with influenza A viruses (IAV) are still amongst the major causes of highly contagious severe respiratory diseases not only bearing a devastating effect to human health, but also significantly impact the economy. Besides vaccination that represents the best option to protect from IAV infections, only two classes of anti-influenza drugs, inhibitors of the M2 ion channel and the neuraminidase, often causing resistant IAV variants have been approved. That is why the need for effective and amply available antivirals against IAV is of high priority. Here we introduce LADANIA067 from the leaves of the wild black currant (*Ribes nigrum folium*) as a potent compound against IAV infections *in vitro* and *in vivo*. LADANIA067 treatment resulted in a reduction of progeny virus titers in cell cultures infected with prototype avian and human influenza virus strains of different subtypes. At the effective dose of 100 µg/ml the extract did not exhibit apparent harming effects on cell viability, metabolism or proliferation. Further, viruses showed no tendency to develop resistance to LADANIA067 when compared to amantadine that resulted in the generation of resistant variants after only a few passages. On a molecular basis the protective effect of LADANIA067 appears to be mainly due to interference with virus internalisation. In the mouse infection model LADANIA067 treatment reduces progeny virus titers in the lung upon intranasal application. In conclusion, an extract from the leaves of the wild black currant might be a promising source for the development of new antiviral compounds to fight IAV infections.

## Introduction

Influenza A viruses (IAV), family members of the genera *Orthomyxoviridae,* are characterised by a negative oriented, segmented single strand RNA genome. Although the natural reservoir of these viruses is within wild living waterfowl, IAV also infect humans and several animal species [1]. As a common cause of highly contagious severe respiratory diseases in humans IAV mainly affect the upper respiratory tract, such as the nose, throat and bronchi [1]. Infections are characterised by the sudden onset of fever, sore throat, headache, severe malaise, muscle pain and nasal inflammation. Thus, IAV not only cause a devastating effect to human health, but also significantly impact the economy. Besides seasonal outbreaks, IAV bear the potential for the development of new pathogenic IAV. The continuous and rapid emergency of new highly pathogenic avian influenza virus (HPAIV) of the H5N1 type, transmitting from poultry to human, and the recent pandemic H1N1v IAV outbreak, to which no vaccines had been amply available in time, highlight the urgent need of effective antivirals to fight influenza viruses. Although, vaccination is the best option to protect from IAV infection, time-consuming generation processes limit its ample availability. Currently two classes of antivirals that either target the M2 protein (amantadine, rimantadine) or the neuraminidase (NA) (oseltamivir, zanamivir) of IAV are available. Unfortunately these compounds are characterised by rapid resistance development. Most H3N2 viruses and human isolates of H5N1 viruses are already resistant to M2-blockers, and an increasing incidence of resistance development against NA-blockers has been reported [2], [3], [4], [5]. While swine-origin influenza viruses (S-OIV) are completely resistant to the adamantanes, interestingly several S-OIV of the 2009 pandemic exhibit also natural resistance to NA-blockers [6], [7], thus bearing potential for multidrug-resistant influenza viruses [8]. Due to the obvious need for new and amply available antiviral agents that do not show any tendency to cause toxic side effects or generate resistant virus variants, the use of plant-derived extracts or compounds received increasing attention. Although various studies have demonstrated the antiviral potential of plant-derived compounds in cell culture, studies that explore their molecular mode of action or their activity *in vivo* are still scarce.

Regarding the active ingredients of plant extracts, a significant antiviral potential has been demonstrated for the group of polyphenols [9]. With more than 8000 phenolic structures, these substances are widely distributed in the plant kingdom. However marginal knowledge on absorption, bio-distribution and metabolism of polyphenols is available. While small polyphenols are absorbed by cells and exhibit antioxidative functions, polymeric polyphenols are only poorly absorbed or metabolized. Numerous studies indicate an antiviral or antibacterial function of polyphenolic substances [9] and antiviral activity against influenza virus infection has been demonstrated *in vitro* and *in vivo* [10], [11].

Recently, CYSTUS052, a polyphenolic substance derived of *Cistus incanus spp. tauricus* has been functionally characterised in context of influenza virus infections. CYSTUS052 directly interferes with the virus particles and thereby blocks virus attachment in a non-specific manner [10], [11]. At the same time cells appear to be inert against the action of the extract ingredients, presumably due to the fact that the active compounds are too big to enter cells and get metabolized. Furthermore, a rather unspecific coverage of the virions themselves lowers the risk of new emerging resistant virus variants. Finally, the effectiveness of this extract against respiratory infections has been already tested in a prospective, randomised, placebo-controlled clinical trial in human patients [12].

Here we focussed on another polyphenol-rich plant, *Ribes nigrum folium* that exhibits broad curative properties [9], [13], [14], [15], [16], [17]. Within the present study we investigated the anti-influenza virus activity of LADANIA067 that is an extract made from the leaves of a wild form of *Ribes nigrum* (black currant). The plant is cultivated but also grows in the wild across central and eastern Europe. It is part of the shrub layer in the level and hill country. *Ribes nigrum folium* is rich in phenolic acids, flavonoids and carotenoids [18]. Furthermore, *Ribes nigrum folium* also contain high amounts of Proanthocyanidins, especially Prodelphinidins, and ascorbic acid [19]. Until today antiviral and antibacterial properties of *Ribes nigrum* fruits, which were used in the past as juice against the common cold, have been described [15]. The yield of anti-oxidants has been shown to be higher in the leaves than in the berries [18]. Our results revealed a potent antiviral activity of LADANIA067 against IAV *in vitro* and *in vivo* without toxic effects or tendency to induce viral resistance.

## Materials and Methods

### Plant Extracts

LADANIA067 Extract Charge-Numbers: #10/17; 020709/069; 180510/158; 150211/281; 140113/523; 140113/524 and CYSTUS052 #080110/095 were supplied and originally developed by Dr. Pandalis NatUrprodukte GmbH & Co. KG (Glandorf, Germany). The reference extract CYSTUS052 is a preparation of *Cistus incanus spp. tauricus* as described in [10], [11]. LADANIA067 is a preparation from the leaves of a wild form of *Ribes nigrum* (black currant). LADANIA067 was delivered in solution and was solved in sterile cell culture medium from 10 to 200 µg/ml and added directly to the medium or virus-stock.

### Viruses, Cells and Viral Infections

Avian influenza virus A/FPV/Bratislava/79 (FPV) (H7N7), recombinant human influenza virus A/Puerto Rico/8/34 (PR8) (H1N1rec) and pandemic swine origin human influenza virus (S-OIV) A/Nordrhein-Wesfalen/173/2009 (H1N1pan), harboring the oseltamivir resistance mutation NA (H275Y) [20], have been propagated and passaged in Madin Darby canine kidney (MDCK) cells. A prototype isolate of human influenza virus A/Puerto Rico/8/34 (PR8) (H1N1) was grown in 10-days-old embryonated chicken eggs. After incubation at 37°C for 2 days the allantois fluid was harvested and used for infection as described below [21].

For infection cells were washed in PBS, incubated with virus diluted in PBS/BA (PBS containing 0.2% BSA, 1 mM MgCl_2_, 0.9 mM CaCl_2_, 100 U/ml penicillin and 0.1 mg/ml streptomycin) for 30 min at 37°C at the indicated multiplicities of infection (MOI). The inoculum was aspirated and cells were incubated with MEM or DMEM containing 0.2% BSA, 1 mM MgCl_2_, 0.9 mM CaCl_2_ and antibiotics. In the case of infection with H1N1, H1N1rec or H1N1pan medium was supplemented with 2 µg/ml trypsin. MDCK cells were grown in MEM and the human lung epithelial cell line A549 was grown in DMEM, respectively. All media were supplemented with 10% heat-inactivated fetal bovine serum (FBS). For pro-apoptotic or pro-inflammatory stimulation of cells Staurosporine (Sigma) (1 µM, 4 h or 8 h), TNFα (Sigma) (5 ng/ml, 20 min), or human recombinant EGF (R&D systems Minneapolis, USA) (30 ng/ml, 5 min) were directly added into the medium.

### Plaque Titrations and Resistance Assays

Supernatants, collected at the indicated time points, were used to assess the number of infectious particles (plaque titers) in the sample. Briefly, MDCK cells grown to 90% confluence in 6-well dishes were washed with PBS and infected with serial dilutions of the supernatants in PBS/BA for 30 min at 37°C. The inoculum was aspirated and cells were incubated with MEM/BA (medium containing 0.2% BSA and antibiotics) supplemented with 0.6% Agar (Oxoid), 0.3%DEAE-Dextran (Pharmacia Biotech) and 1.5% NaHCO_3_ at 37°C, 5%CO_2_ for 2–3 days. Virus plaques were visualized by staining with neutral red. The virus titers are presented as plaque-forming units per ml (pfu/ml). Generation of resistant virus variants to antiviral treatment was assessed. Briefly A549 cells were infected with the influenza A virus strain FPV (MOI = 0.001) and were left untreated or treated with the indicated amounts of LADANIA067. Unless otherwise indicated cells and viruses were pre-incubated with LADANIA067 for 30 min and LADANIA067 has been further added to the medium throughout the infection. To evaluate the resistance development 24 h post infection (p.i.) supernatants were taken and used for a second infection round. This procedure was repeated for several subsequent passages. As a reference antiviral compound cells have been treated with amantadine (Sigma) (5 µM) that is know to induce IAV resistance quite rapidly. Supernatants were assayed for progeny virus yields by standard plaque titrations. Virus yields of mock-treated cells were arbitrarily set as 100%.

### MTT-cell Proliferation Assay

The MTT [3-(4,5-dimethylthiazol-2-yl)-2,5-diphenyltetrazoliumbromide] assay is based on an enzymatic reaction of the mitochondrial succinic dehydrogenase. In viable and proliferating cells these enzyme cleaves the tetrazolium rings of the pale yellow MTT which results in formation of dark blue formazan that is largely impermeable to cell membranes and therefore accumulates in healthy cells. The amount of formed formazan can be measured in a colorimetric assay at OD 562 nm and is directly proportional to the number of proliferating cells. A549 cells were left untreated or treated with the indicated amounts of LADANIA067 for different time periods. Afterwards cells were washed with PBS and incubated with MTT (5 mg/ml) for 3 hours at 37°C. Reaction was blocked by DMSO (Sigma) and cells were further incubated for 20 min at 37°C. The colour reaction was measured in an Emax precision microplate reader at 562 nm. The untreated control was arbitrarily set as 100%.

### Plasmids, Transient Transfection and Reporter Gene Assay

A constitutive active CMV promoter luciferase plasmid was transfected with Lipofectamine 2000 (Invitrogen) into A549 cells according to a protocol by Basler et al. [22]. Eight hours post transfection cells were left untreated or treated with the indicated amounts of LADANIA067 or 10 µg/ml Cycloheximide (BioTrend) for 18 hours. Subsequently cells were harvested with 200 µl lysis buffer [50 mM Na-MES, pH 7.8, 50 mM Tris-HCl, pH 7.8, 10 mM dithiothreitol (DTT) and 2% Triton X-100]. Luciferase activity was measured and given as relative fold activation ±SD of nine samples as the ratio of luciferase activity relative to the DMSO control.

### Flow Cytometry Analysis

A549 cells were left untreated or treated with LADANIA067 of the amounts and for times indicated. Cells were washed with PBS, trypsinized, washed again and resuspended in PBS containing propidium iodide (PI) in a concentration of 50 µg/ml. After incubation for 1 h at room temperature (RT) PI was removed by washing cells with PBS and fluorescence was determined in the FL2-channel (585 nm) using a FACScalibur cytometer (Becton Dickinson). As positive control A549 cells were treated with Staurosporine (1 µM) and EDTA (5 mM) for 4 h and analysed as described above.

### Western Blotting

For Western blots cells were lysed on ice with RIPA lysis buffer (1% (v/v) NP-40, 0.5% (v/v) DOC, 1% (w/v) SDS, 150 mM NaCl, 50 mM Tris pH 8, 90% H_2_O dest., 200 µM pefablock, 5 µg/ml aprotinin, 5 µg/ml leupeptin, 1 mM sodium-vanadate, 5 mM benzamidine) for 30 min. Cell lysates were cleared by centrifugation and protein yields were estimated employing a protein dye reagent (Bio-Rad Laboratories). Equal amounts of protein were separated by SDS-polyacrylamide gel electrophoresis and subsequently blotted on nitrocellulose membranes. Anti-PARP monoclonal antiserum was purchased from BD Transduction Laboratories. Antiserum against the influenza virus protein M1 was obtained from ABSerotec. Antisera against phosphospecific EGFR(Y1068), EGFR and phosphospecific ERK1/2 (pT202/pY204, pT185/pY187) were purchased from Cell Signaling Technology. Antiserum against IκBα was purchased from Santa Cruz Biotechnologies. Loading controls were performed with ERK2 antiserum (Santa Cruz Biotechnologies). Protein bands were visualized in a standard enhanced chemiluminescence reaction.

### Hemagglutination-inhibition-test

Influenza viruses are characterised by their ability to agglutinate erythrocytes. This hemagglutinating activity can be visualized upon mixing virus dilutions with chicken erythrocytes in microtiter plates. The chicken erythrocytes (Lensing-Baeumer, Ibbenbueren) supplemented with 1.6% sodium citrate (Sigma) in sterile water were separated by centrifugation (800×g, 10 min, room temperature) and washed three times with sterile PBS (Invitrogen). The lowest amount of virus particles able to agglutinate the chicken erythrocytes was determined in a serial virus dilution and used to investigate the inhibitory effect of LADANIA067 in comparison to CYSTUS052 onto the hemagglutinating activity. Untreated erythrocytes precipitate to the bottom of the plate, while upon preincubation with virus the blood cells show an even and diffuse distribution. Briefly, LADANIA067 and CYSTUS052 were serially diluted as indicated. From virus stock (H7N7, 1×10^9^ pfu) 1/128 dilutions were made, and 50 µl/well of this virus dilution was added as indicated. After preincubation of 45 min, chicken erythrocytes (1/20 in PBS) were mixed with the solution.

### Binding Analysis of LADANIA067 to Cellular Surface and Indirect Immunofluorescence Microscopy

A549 cells were left untreated or treated with LADANIA067 (100 µg/ml) for 1 h at 37°C. Subsequently biotinylated *Sambucus nigra* agglutinin (SNA) purchased from Linaris (Wertheim, Germany), (1 µg/ml) was incubated for 1 h at 4°C with A549 cells. SNA recognizes α-2–6 Galactosidases, predominantly present at the surface of A549 cells and responsible for IAV binding of human species. Subsequently cells were incubated with Cy3-conjugated streptavidin to detect lectins and were analysed in fluorescence microscopy. Fluorescence was visualized using a Axiovert 2000 ApoTome microscope with AxioCam digital camera and AxioVision software (Zeiss). To monitor cell morphology transmitted light microscopy was applied, using the same equipment.

### *In vivo* Experiments

All animal studies were performed in compliance with animal welfare regulations (permission number Az 8.87–50.10.36.09.007 by the State Agency for Nature, Environment and Consumer Protection [LANUV], Germany).

Eight-week-old BALB/c mice were obtained from the animal-breeding facilities Harlan-Winkelmann. For infection, the animals were anesthetized by intraperitoneal injection of 200 µl solution of ketamine (Ceva) and xylazin (Ceva; equal amounts of 2% xylazin solution and 10% ketamine solution were mixed 1∶10 with PBS). Mice were treated with LADANIA067 using the COAALA Mouse Aerosol Application System (Activaero GmbH). The LADANIA067 extract was dissolved in sterile H_2_O and stock solutions of 10 mg LADANIA067/ml and 15 mg LADANIA067/ml were prepared. BALB/c mice were placed in the tube-cylinder and exposed to 1.5 ml (per mouse), 2 bar of aerosolized LADANIA067 extract for 10 min twice a day for three (lung titer) or five days (body weight). The control group was treated with the same amount of H_2_O correspondingly. Mice were infected ten minutes after the first exposure via the intranasal route with the influenza virus strain A/FPV/Bratislava/79 (H7N7) (10^3^ pfu) or the recombinant influenza virus A/Puerto Rico/8/34 (H1N1rec) (4×10^2^ pfu) in a 50 µl volume to determine the viral replication in the lung or A/FPV/Bratislava/79 (H7N7) (5×10^2^ pfu) to monitor the body weight. The general health status of the animals was controlled twice a day and body weight was measured every day. Due to protection of animal welfare restrictions, mice were sacrificed upon a body weight loss of 20% at the utmost. For determination of lung virus titer, mice lungs were collected in 3 ml PBS at day 3 p.i. The samples were homogenized using a FastPrep24 homogenizator (MP Biomedicals) with Lysing Matrix D (MP Biomedicals). The samples were centrifuged at 10 000 rpm for 10 min at 4°C. The supernatants were taken and assayed in standard plaque analysis as described above.

## Results

### LADANIA067 Inhibits Influenza A Virus Replication in a Concentration Dependent Manner

**Figure 1:**
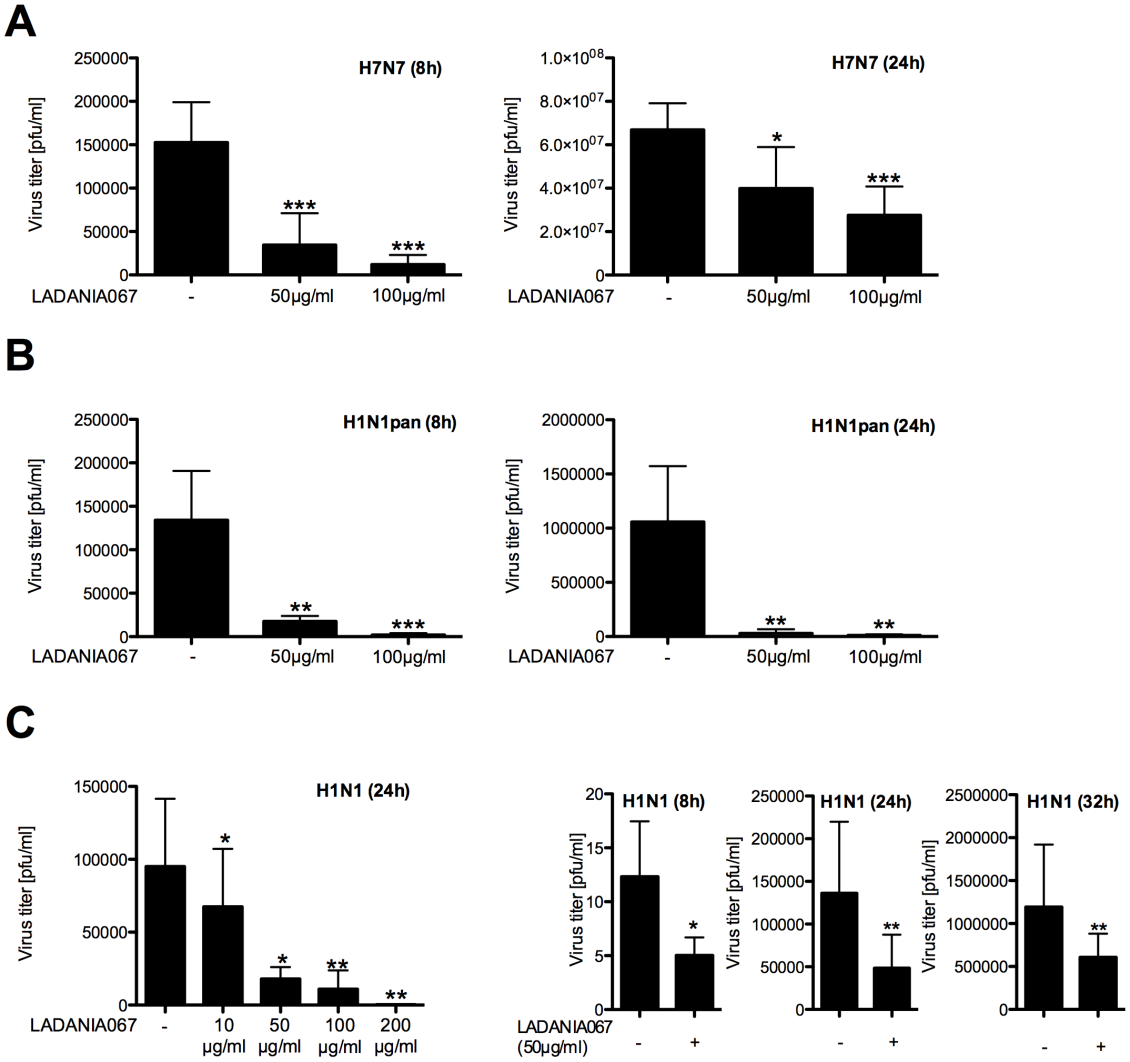
LADANIA067 impairs IAV replication. A549 cells were left untreated or treated with the indicated amounts of LADANIA067 before and during infection with the influenza virus strains A/FPV/Bratislava/79 (FPV) (H7N7) (8 h: MOI = 0.1; 24 h: MOI = 0.01) (A), A/NRW/173/09, an pandemic swine-origin-IAV (SO-IAV) (H1N1pan) (8 h: MOI = 1.5; 24 h: MOI = 0.5) (B) or A/Puerto Rico/8/34 (PR8) (H1N1) (MOI = 0.01) (C). In (C) cells were left untreated or treated with the indicated amounts (left panel) or 50 µg/ml of LADANIA067 before and during infection. (A–C) The virus was pretreated with LADANIA067 as well. Supernatants were assayed for progeny virus particles at the times indicated. Data represent means ±SD of 4 biological samples. Statistical significance was assessed by students t-test (*p<0.05, **p<0.01, ***p<0.001).

In a first set of experiments we investigated whether LADANIA067 exerts an antiviral effect against influenza A virus (IAV) infection in cultured cells. Therefore, the commonly used human alveolar type II epithelial cell line A549 was incubated with LADANIA067 at various concentrations for 30 min before infection and during the on-going infection process. Additionally, the virus was pre-incubated with LADANIA067 at the various concentrations for 30 min prior to infection (Fig. 1A–C). For infection we used different IAV strains, including the highly pathogenic avian H7N7 influenza virus A/FPV/Bratislava/79 (FPV) (Fig. 1A), an oseltamivir-resistant variant of swine-origin H1N1 influenza virus, strain A/Nordrhein-Westfalen/173/09 (H1N1pan) (Fig. 1B) and the human H1N1 prototype isolate A/Puerto-Rico/8/34 (PR8) (H1N1) (Fig. 1C). For all three viruses a dose-dependent reduction of progeny virus titers was detected upon LANDANIA067 treatment in single-cycle (8 h) and multi-cycle (24 h, 32 h) experiments indicating a broad antiviral activity towards different IAV strains.

### LADANIA067 Treatment does not Affect Cell Morphology and Viability and does not Negatively Interfere with Cellular Proliferation and Metabolism

**Figure 2:**
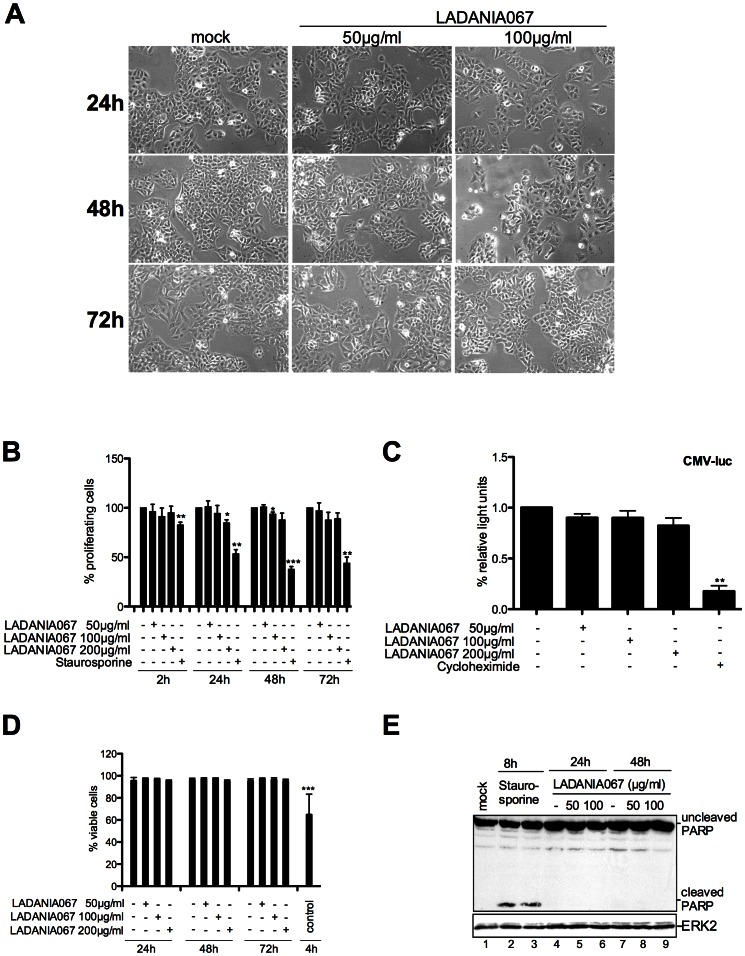
LADANIA067 is not toxic or proapoptotic in A549 cells. (A) A549 cells were left untreated or treated with LADANIA067 with the amounts and times as indicated. The impact of LADANIA067 on cell morphology was visualized by an Axiovert 2000 ApoTome microscope (100-fold magnification) and monitored by photography. (B) A549 cells were left untreated or treated with 50 µg/ml, 100 µg/ml or 200 µg/ml LADANIA067 or 1 µM Staurosporine for the indicated time periods. Cell proliferation/viability was estimated via colorimetric assay that followed incubation of cells with MTT. The proliferating cells are depicted, whereby the untreated controls were arbitrarily set as 100%. Data represent ±SD of twelve biological samples. (C) A549 cell were transfected with a constitutive active CMV promoter luciferase plasmid. Eight hours post transfection cells were left untreated or treated with the indicated amounts of LADANIA067 or 10 µg/ml Cycloheximide respectively for 18 hours. Data represent ±SD of nine biological samples and are given as the ratio of luciferase activity relative to the untreated control. (D) A549 cells were incubated in the presence and absence of 50 µg/ml, 100 µg/ml or 200 µg/ml LADANIA for 24 h, 48 h and 72 h. A549 cells that were treated with Staurosporine (1 µg/ml) in addition to EDTA (5 mM) for 4 hours served as control. Percentage of viable cells was measured by staining of dead cells with propidium iodide (PI) as described in *Materials and Methods.* Data represent ±SD of nine biological samples. Statistical significance was assessed by students t-test (*p<0.05, **p<0.01, ***p<0.001) (B, C, D). (E) A549 cells were left untreated or were treated with 1 µM Staurosporine for 8 h or 50 µg/ml or 100 µg/ml LADANIA067 for 24 h or 48 h. Upon stimulation cell lysates were prepared and subjected to Western blot, and caspase mediated PARP cleavage was determined as a measure for apoptosis induction. ERK2 expression was monitored as loading control.

Since drug safety is a major issue for antiviral agents, we analyzed whether antiviral acting concentrations of LADANIA067 would have any harming effect on healthy cells (Fig. 2A). Initially, cells were treated with LADANIA067 (50 µg/ml and 100 µg/ml) for different time periods (up to 72 h) and the morphology of untreated or LADANIA067-treated cells was compared. No difference in cell shape or cell numbers could be observed (Fig. 2A). Further, MTT cell proliferation assays revealed similar cell proliferation independent of the presence or absence of LADANIA067 (Fig. 2B). Also, the observed reduction in progeny virus titers was not due to a general inhibition of cellular transcription and translation because constitutive expression of a reporter gene (luciferase) driven by a constitutively active promoter (CMV) was not affected by LADANIA067 treatment in contrast to the Cycloheximide control (Fig. 2C). In a cytotoxicity assay, A549 cells were treated with LADANIA067 for different time periods (up to 72 h) and were stained with propidium-iodide (PI) to monitor cell death (Fig. 2D). Again, no significant changes in the number of dead cells could be detected. In accordance with these data, LADANIA067 did not induce apoptosis as evidenced by a lack of cleavage of Poly-ADP-ribose-polymerase (PARP), a prominent substrate of apoptotic caspases (Fig. 2E). In conclusion, the extract did not show any significant harmful effects on cell proliferation or cell survival, and did not alter transcription or translation in this system.

### An Early Step in the Infection Process is Blocked Upon Treatment of Cells with LADANIA067

**Figure 3:**
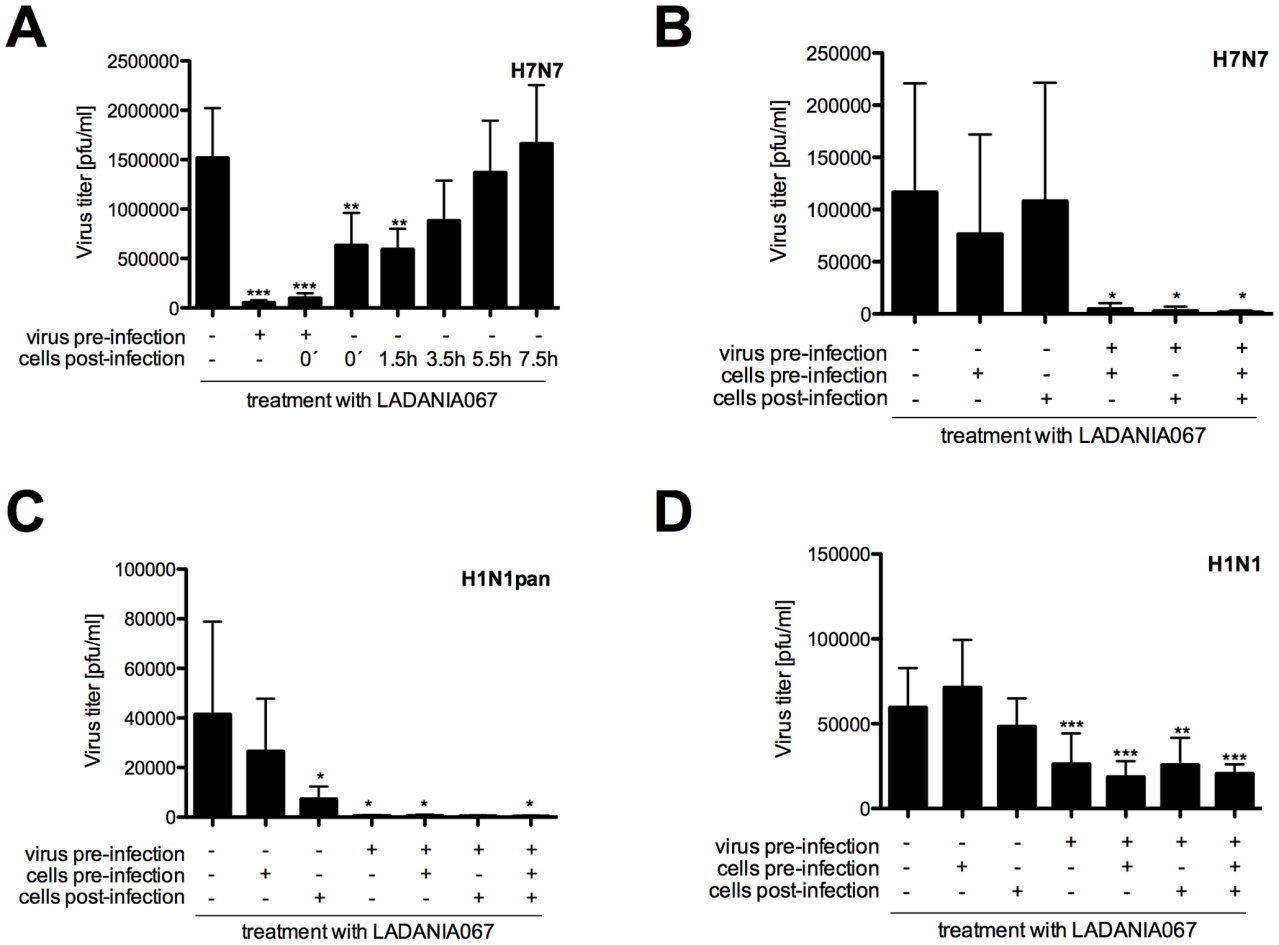
LADANIA067 inhibits IAV replication at early times of infection. (A) A549 cells were left untreated or treated with 100 µg/ml of LADANIA067, which was added at the indicated times during infection with FPV (H7N7) (MOI = 1). (B-D) A549 cells and/or virus were left untreated or treated with 100 µg/ml of LADANIA067 pre-infection or post-infection (p.i.). For infection FPV (H7N7) (MOI = 0.1), SO-IAV (H1N1pan) (MOI = 1) and PR8 (H1N1) (MOI = 5) have been used. Supernatants were assayed for progeny virus particles 8 h p.i. Data represent means ±SD of 4 biological samples (A) or 6 biological samples (B–D). Statistical significance was assessed by students t-test (*p<0.05, **p<0.01, ***p<0.001).

To determine the step in the IAV life cycle that is affected by LADANIA067, supernatants from cells that were treated with LADANIA067 at different time points pre- and post-infection were analyzed for their content of progeny virus (Fig. 3). Interestingly, most prominent reductions of virus titers were obtained when the infectious virus was pretreated with LADANIA067, whereas effects were far less pronounced when LADANIA067 was added exclusively to cells pre- or post-infection.

**Figure 4:**
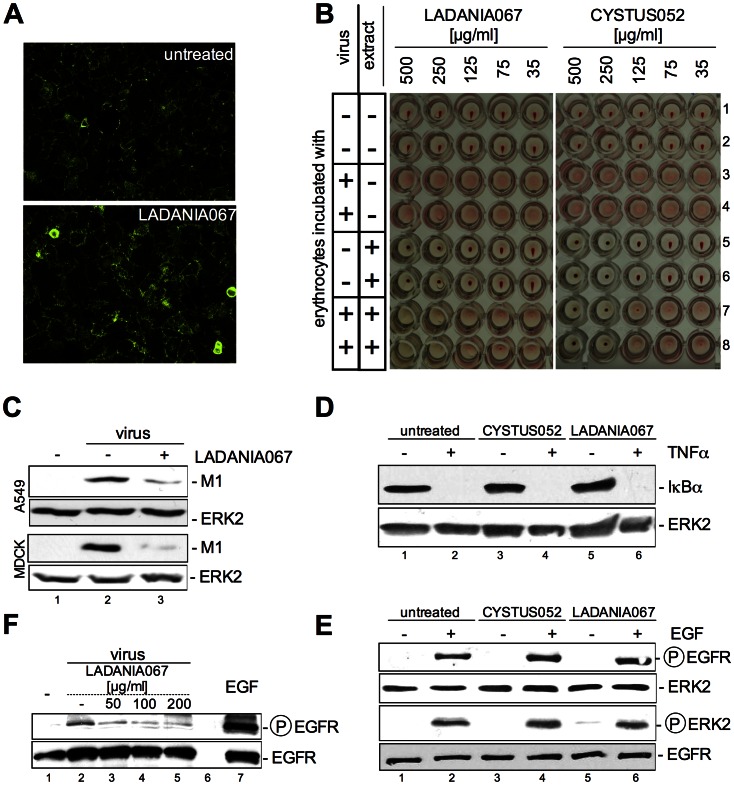
LADANIA067 inhibits viral internalisation. (A) A549 cells were left untreated or treated with 100 µg/ml LADANIA067 for 1 h at 37°C. Biotinylated *Sambucus nigra* agglutinin (SNA) (1 µg/ml) was added and cells were incubated for 1 h at 4°C with A549 cells. Subsequently, cells were incubated with Cy3-conjugated streptavidin to detect lectins for fluorescence microscopy using an Axiovert 2000 ApoTome microscope with AxioCam digital camera and AxioVision software (Zeiss) (20-fold magnification) (B) LADANIA067 was diluted as indicated. Influenza A/FPV/Bratislava/79 was diluted in PBS (4×10^5^ pfu/well) and 50 µl was added per well of a 96-well plate. After preincubation of 45 min, chicken erythrocytes (1∶20 in PBS) were mixed with the solution. In samples where viruses were preincubated with LADANIA067 or CYSTUS052, up to a certain dilution the virus particles are no longer capable of agglutinating erythrocytes, indicating an interaction of LADANIA067 or CYSTUS052 with the viral HA. (C) A549 cells (upper panel) or MDCK cells (lower panel) were left untreated or treated with 200 µg/ml LADANIA067 30 min before infection. Also the influenza virus A/Puerto Rico/8/34 was left untreated or was incubated with the same amount of LADANIA067 at 4°C for 30 min. Subsequently cells were left untreated or infected (MOI = 10) with virus for 30 min at 4°C till a temperature shift to 37°C for further 15 min. Upon stimulation cells were washed twice with PBS and cell lysates were prepared and subjected to Western blot. Viral M1 protein was determined as a measure for attached and internalized virus particles. A549 cells were left untreated or treated with 100 µg/ml LADANIA067 for 4 h prior to stimulation with (D) 5 ng/ml TNFα for 20 min or (E) 30 ng/ml EGF for 10 min. (F) Influenza virus A/Puerto Rico/8/34 was left untreated or was incubated with the indicated amounts of LADANIA067 supplemented with medium for 30 min at 4°C. Subsequently, cells were washed with PBS and incubated for 60 min at 37°C without virus or with virus which was left untreated or was pretreated with LADANIA067. Upon stimulation cell lysates were prepared and subjected to Western blot. (D) Expression of IκBα, (E, F) phosphorylation of EGFR and ERK2 were detected and (D, E, F) EGFR or ERK2 expression was monitored as loading control.

The earliest event during the infection cycle is virus binding to sialic-acid residues at the cellular surface. To analyse whether LADANIA067 may interfere with sialic-acids of cellular surface-proteins and thus prevents viral attachment, we made use of lectin binding assays. Lectins bind sialic acids in a multivalent fashion and cause hemagglutination similar to IAV [23]. The lectin *Sambucus nigra* agglutinin (SNA) has a preference for 2–6 coupled sialic acids (Siaα2–6Gal), which are present on A549 cells [24]. Interestingly, upon treatment of cells with LADANIA067 lectin binding was not reduced (Fig. 4A) indicating that the extract did not hinder the accessibility of the receptor for IAV on the cell surface.

Since our data indicated an inhibitory effect of LADANAIA067 very early in the infection process, we further investigated whether the compound directly interferes with the virus particle itself to inhibit infection. In a hemagglutination inhibition (HI) assay (Fig. 4B) we made use of the agglutinating feature of IAV towards red blood cells (RBC). IAV-binding of the hemagglutinin provokes crosslinking of RBC and will form a type of lattice in this case. This results in a diffuse appearance of the RBC in a round-bottom vial (Fig. 4B lanes 3 and 4) in contrast to a spot like appearance of precipitated RBC in the absence of any virus or other agglutinating agent (Fig. 4B lanes 1 and 2). We used the plant extract CYSTUS052 as a control that already has previously been shown to prevent binding of IAV to RBC [10]. Accordingly, CYSTUS052 showed inhibition of RBC agglutination at concentrations of 250 µg/ml and 500 µg/ml (Fig. 4B right panel lanes 7 and 8). In contrast, LADANIA067 was not able to inhibit RBC agglutination at all, arguing against physical interaction of the extract ingredients with the virion that would prevent binding to cells (Fig. 4B left panel lanes 7 and 8). Nonetheless, to further confirm that IAV replication is inhibited at a very early stage of infection by LADANIA067 treatment, we visualized IAV internalisation by detection of virion-associated matrix protein (M1), the most abundant viral protein in IAV particles, in Western blot analysis (Fig. 4C). This approach has been previously described to be a quite selective assay to monitor virus uptake [25]. M1 protein levels were reduced in LADANIA067 treated samples, indicating inefficient viral uptake (Fig. 4C). This correlated well with reduced progeny virus titers in cases of pre-treated virus samples (Fig. 3).

Thus, our results clearly indicate that LADANIA067 inhibits virus internalisation, but at the same time does not interfere with cellular viability, metabolism or transcription. Nonetheless, to further assess whether LADANIA067 may provoke any other pharmacological effect in cells, e.g. activation of intracellular signalling events, we monitored the activation of different signalling pathways upon stimulation with tumor necrosis factor α (TNFα) or epidermal growth factor (EGF). The cytokine TNFα is a strong activator of the cellular transcription factor NF-κB (Fig. 4D, lanes 2, 4, 6). Activation of NF-κB was monitored in Western blots by examination of degradation of IκBα, the inhibitor of NF-κB (Fig. 4D, upper panel). In TNFα stimulated cells IκBα was fully degraded, independent whether they were left untreated or were pre-treated with LADANIA067 (Fig. 4D, upper panel, lanes 5, 6) or CYSTUS052 (Fig. 4D, upper panel, lanes 3, 4). This indicates that LADANIA067 treatment neither activates NF-κB nor inhibits receptor-mediated NF-κB activation. The epidermal growth factor receptor (EGFR), a receptor tyrosine kinase, is another important cellular signalling inducer, which has also been identified to regulate IAV internalisation [24]. Thus, we also monitored whether LADANIA067 may interfere with EGFR signalling in the presence or absence of EGF. A549 cells were treated with LADANIA067 or were left untreated (Fig. 4E) prior to stimulation with EGF. We also included a reference extract in these assays, CYSTUS052 that was previously shown to fail in activating or inhibiting receptor-mediated signalling events [10]. EGF-mediated activation of EGFR and the downstream signalling kinase ERK2 were monitored in Western blots with activation-state specific anti-phospho-site antibodies. In these assays LADANIA067, like CYSTUS052 failed to affect phosphorylation of EGFR or ERK2, regardless whether the cells were pretreated with one of the compounds or not. In summary, our results indicate that cells are inert to LADANIA067 with respect to TNFα or EGF-induced signalling responses. These results may stand as general examples for cellular ligand/receptor systems. It was already shown that IAV also activate the EGFR itself during initial binding of IAV particles to cellular surfaces. If LADANIA067 is able to block the interaction of IAV particles with host cells as assumed, IAV induced EGFR activation should be suppressed. Indeed, IAV induced EGFR activation was blocked in the presence of LADANIA067 (Fig. 4F), verifying the interfering function of LADANIA067 with virus particles itself that in turn inhibits IAV internalisation into the host cells.

### LADANIA067 does not Show any Tendency to Induce Viral Resistance

**Figure 5:**
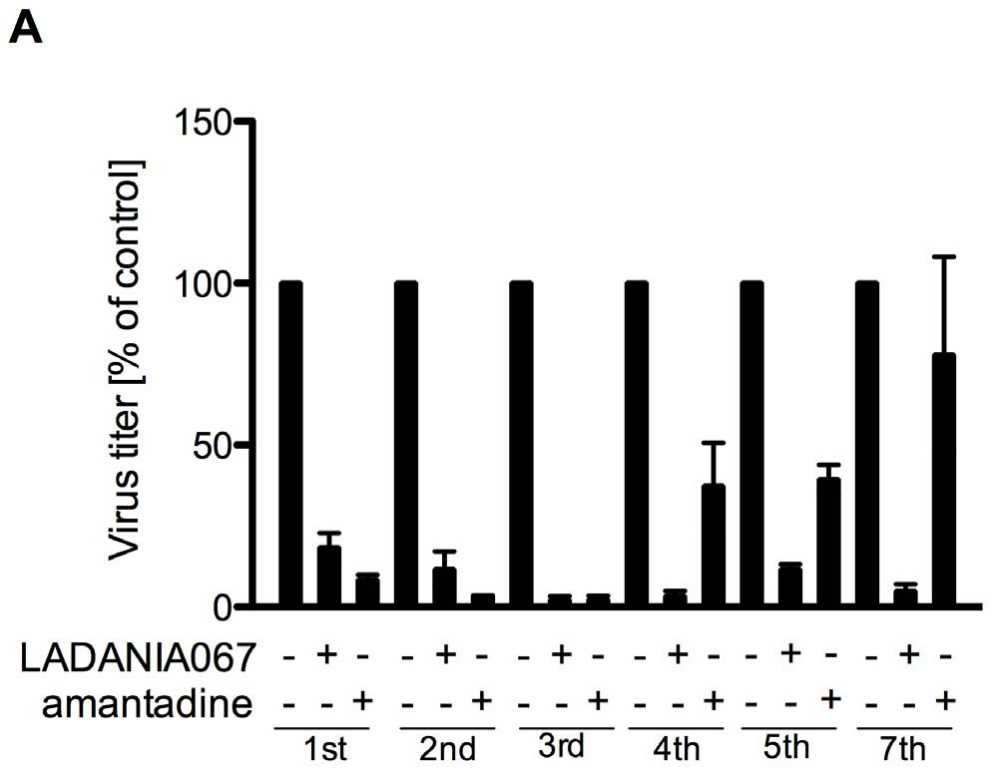
LADANIA067 shows no tendency to induce virus drug resistance. A549 cells were infected with FPV (MOI = 0.001) for 24 h and left untreated or treated with 100 µg/ml of LADANIA067 or 5 µM amantatine. Cells and virus were pre-incubated with LADANIA067 for 30 min and LADANIA067 has been added to the medium throughout the infection. Amantadine was added to the medium upon virus inoculation of the cells. The supernatants were taken and employed for infection in the next round of investigation.

Resistance to currently licenced anti-influenza drugs is an increasing problem in clinical use. Thus, we explored whether continuous LADANIA067 treatment would provoke emergence of resistant variants in an established multi-passaging experiment. The results revealed that LADANIA067 treatment did not result in the development of resistant virus variants (Fig. 5). LADANIA067 still efficiently blocked IAV propagation after the 5^th^ to 7^th^ passage when viral replication capacity in amantadine treated cells was fully back to the untreated situation. In that respect LADANIA067 resembles the reference extract CYSTUS052 that also blocked virus internalisation and did also not cause IAV resistance in a similar experimental setting [10].

### LADANIA067 Exhibits Antiviral Functions *in vivo*

**Figure 6:**
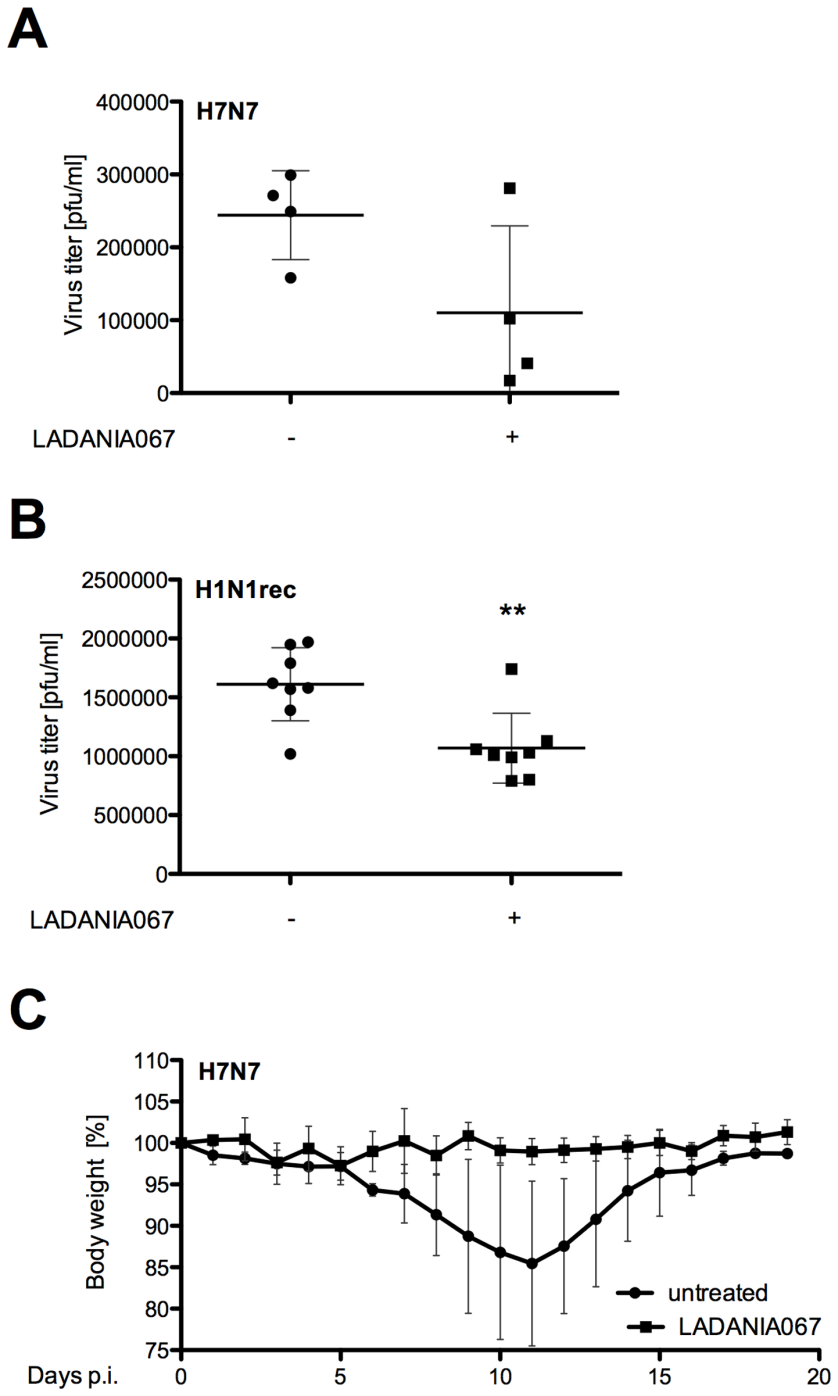
LADANIA067 efficiently blocks influenza virus propagation *in vivo*. (A, C) Four 8-week-old BALB/c mice per group were intranasally infected with sublethal doses (A) 10^3^ pfu or (C) 5×10^2^ pfu of FPV (H7N7). (B) Eight 8-week-old BALB/c mice per group were intranasally infected with sublethal doses of recombinant PR8 (H1N1rec) (4×10^2^ pfu). Mice were either solvent or LADANIA067 treated (1.5 ml per mice, at a concentration of (A, C) 10 mg/ml or (B) 15 mg/ml) via inhalation, twice a day for (A, B) three days or (C) five days. (A, B) Virus titers of infected lungs were determined 3 days p.i. in standard plaque titrations. (C) Body weight of the infected mice was monitored.

Based on our results, LADANIA067 may be a promising source for an antiviral agent that does not exhibit harmful side effects on cell viability or metabolism and does not show any tendency to induce resistant virus variants. Nonetheless, antiviral activity in *in vitro* experiments may be different *in vivo*. Thus, we tested the antiviral activity of LADANIA067 *in vivo* in BALB/c mice infected with a sublethal dose of influenza virus A/FPV/Bratislava/79 (H7N7). With regard to cleavability of its hemagglutinin as a major determinant of pathogenicity this strain belongs to highly pathogenic avian influenza virus (HPAIV), a group also including the H5N1 “bird-flu” viruses. Since the active ingredients of LADANIA067 do not seem to be taken up to cells or get metabolized, we favoured a topical rather than a systemic treatment and therefore applied LADANIA067 directly into the mouse lung as an aerosol. Four BALB/c mice were treated for three (Fig. 6A) or five (Fig. 6C) consecutive days with 1.5 ml LADANIA067 extract (10 mg/ml) twice a day using the COAALA Mouse Aerosol Application System (Activaero GmbH). As control four infected BALB/c mice were treated with the solvent H_2_O. Reduction of virus titers in the lung of infected animals was detectible at day three of infection (Fig. 6A, B). The differences in virus accumulation in lungs correlated well with the body weight of the animals (Fig. 6C). In comparison to H_2_O treated IAV infected BALB/c mice showing a clear body weight loss whereas LADANIA067 treated, did not show any body weight reduction. In an additional *in vivo* experiment (Fig. 6B) eight BALB/c mice per group were infected with recombinant influenza virus A/Puerto Rico/8/34 (H1N1rec). Treatment for three consecutive days with 1.5 ml LADANIA067 extract (15 mg/ml) in comparison to the solvent H_2_O control twice a day using the COAALA Mouse Aerosol Application System (Activaero GmbH), resulted in significantly reduced viral lung titers upon LADANIA067 treatment, verifying our former results.

## Discussion

Infections with IAV are still major plagues worldwide with the potential to cause devastating pandemics. The increasing frequencies of viral resistance to the currently available antivirals that exclusively target specific viral factors highlight the importance for novel antiviral strategies. In the present study we demonstrate that an extract derived from *Ribes nigrum folium*, LADANIA067, exerts a potent antiviral activity *in vitro* and *in vivo.* On a molecular basis the extract appears to interfere with the virus particle, preventing virus internalisation. However, unlike the reference extract CYSTUS052, LADANIA067 does not interfere with the viral hemagglutinin, since viral agglutination of RBC was not affected in the presence of antiviral concentrations of LADANIA067. Furthermore, LADANIA067 does not directly interact with sialic acids (SA), the cellular receptors of influenza viruses. Nonetheless, an interaction with other cell surface components, such as proteins and oligosaccharides can not be ruled out even if enhanced binding to the cellular surface should have been impeded some of the cellular functions examined or binding of ligands to the cell surface, as analyzed in Fig. 4D, E.

An integral feature of LADANIA067 is the very high content in polyphenols, including gallic acid and *p*-hydroxybenzoic acid [18]. Polyphenols bear protein-binding capacity, probably also physically interacting with pathogens and/or cellular surfaces in an unspecific manner. In fact LADANIA067 seems to interfere with the virus particles during virus internalisation as indicated by a lack of IAV induced EGFR phosphorylation in the presence of LADANIA067. Concomitantly signal induction during virus internalisation is prevented resulting in reduced viral uptake into the host cells. Another major evidence of an unspecific rather than a specific interference of LADANIA067 with the virus particle may be the reduced capability to develop resistant variants. Additionally, polyphenols have been demonstrated to possess antibacterial activity as well, thus also acting against bacterial co-infections, another threatening complication in severe influenza virus infection [15]. Although to date the nature of the active ingredient(s) of LADANIA067 is not known and, dependent on the extraction method variations can occur [13], [18], the identified action profile points to the high polyphenol content as being responsible for its antiviral activity.

LADANIA067 treatment did not result in negative effects on cell proliferation, metabolism, transcriptional/translational activity or the responsiveness of the cell to ligands. Especially the latter is indicative of a missing pharmacological activity of LADANIA067, which is probably due to the fact that the active ingredients of the extract are not taken up to cells. However, this is also a disadvantage with regard to treatment options, since LADANIA067 has to be applied locally as an antiviral agent. In fact, our first results indicate that LADANIA067 is a potent antiviral agent *in vivo,* since aerosol treatment of infected mice protected these animals from the disease.

Although LADANIA067 is not able to prevent RBC agglutination by IAV in comparison to CYSTUS052, several functional similarities exist between the two compounds. Moreover, these kinds of plant extracts may also be considered for prophylaxis. Fruits, leaves and buds of this plant have been used for centuries in traditional cuisine and even traditional medicine, without reports of side effects or allergic reactions. Further, the emergency of resistant virus variants is lowered due to a putative unspecific interaction with the pathogens. Such an unspecific interaction with the pathogen that prevents binding to cellular receptors greatly rules out an induction of antiviral immune responses. This is in contrast to viral interference with other receptor-mediated processes, e.g. engagement of receptors of the proteinase-activated receptor family (PAR) that act via enhancement of IFNγ-production [26], [27], [28].

In particular, the inhalable formulation of LADANIA067 shows potential as a replacement or supplementary strategy to current anti-influenza therapeutics.

Although the efficacy of LADANIA067 action in infected individuals has still to be confirmed, this plant extract holds promising antiviral effects and may serve as novel amply available anti-influenza drug.

## References

[1] Wright PF, Neumann G, Kawaoka Y (2007) Orthomyxoviruses. In: Knipe DM, Howley PM, editors. Fields Virology. 5 ed. Philadelphia, PA 19106 USA: LIPPINCOTT WILLIAMS & WILKINS, a WOLTERS KLUWER BUSINESS. 1691–1740.

[2] Tosh C, Murugkar HV, Nagarajan S, Tripathi S, Katare M, et al (2011) Emergence of amantadine-resistant avian influenza H5N1 virus in India. Virus Genes 42: 10–15. 20953687 10.1007/s11262-010-0534-z

[3] Puthavathana P, Auewarakul P, Charoenying PC, Sangsiriwut K, Pooruk P, et al (2005) Molecular characterization of the complete genome of human influenza H5N1 virus isolates from Thailand. J Gen Virol 86: 423–433. 15659762 10.1099/vir.0.80368-0

[4] Le QM, Kiso M, Someya K, Sakai YT, Nguyen TH, et al (2005) Avian flu: isolation of drug-resistant H5N1 virus. Nature 437: 1108. 16228009 10.1038/4371108a

[5] Boltz DA, Douangngeun B, Phommachanh P, Sinthasak S, Mondry R, et al (2010) Emergence of H5N1 avian influenza viruses with reduced sensitivity to neuraminidase inhibitors and novel reassortants in Lao People’s Democratic Republic. J Gen Virol 91: 949–959. 20016036 10.1099/vir.0.017459-0 PMC2888158

[6] Hurt AC, Chotpitayasunondh T, Cox NJ, Daniels R, Fry AM, et al (2012) Antiviral resistance during the 2009 influenza A H1N1 pandemic: public health, laboratory, and clinical perspectives. Lancet Infect Dis 12: 240–248. 22186145 10.1016/S1473-3099(11)70318-8

[7] Bearman GM, Shankaran S, Elam K (2010) Treatment of severe cases of pandemic (H1N1) 2009 influenza: review of antivirals and adjuvant therapy. Recent Pat Antiinfect Drug Discov 5: 152–156. 20334616 10.2174/157489110791233513

[8] van der Vries E, Schutten M, Boucher CA (2011) The potential for multidrug-resistant influenza. Curr Opin Infect Dis 24: 599–604. 22001947 10.1097/QCO.0b013e32834cfb43

[9] Daglia M (2011) Polyphenols as antimicrobial agents. Curr Opin Biotechnol 23: 174–181. 21925860 10.1016/j.copbio.2011.08.007

[10] Ehrhardt C, Hrincius ER, Korte V, Mazur I, Droebner K, et al (2007) A polyphenol rich plant extract, CYSTUS052, exerts anti influenza virus activity in cell culture without toxic side effects or the tendency to induce viral resistance. Antiviral Res 76: 38–47. 17572513 10.1016/j.antiviral.2007.05.002

[11] Droebner K, Ehrhardt C, Poetter A, Ludwig S, Planz O (2007) CYSTUS052, a polyphenol-rich plant extract, exerts anti-influenza virus activity in mice. Antiviral Res 76: 1–10. 17573133 10.1016/j.antiviral.2007.04.001

[12] Kalus U, Grigorov A, Kadecki O, Jansen JP, Kiesewetter H, et al (2009) Cistus incanus (CYSTUS052) for treating patients with infection of the upper respiratory tract. A prospective, randomised, placebo-controlled clinical study. Antiviral Res 84: 267–271. 19828122 10.1016/j.antiviral.2009.10.001

[13] Tabart J, Kevers C, Pincemail J, Defraigne JO, Dommes J (2006) Antioxidant capacity of black currant varies with organ, season, and cultivar. J Agric Food Chem 54: 6271–6276. 16910719 10.1021/jf061112y

[14] Knox YM, Suzutani T, Yosida I, Azuma M (2003) Anti-influenza virus activity of crude extract of Ribes nigrum L. Phytother Res. 17: 120–122. 10.1002/ptr.1053 12601672

[15] Ikuta K, Hashimoto K, Kaneko H, Mori S, Ohashi K, et al (2012) Anti-viral and anti-bacterial activities of an extract of the Blackcurrant (Ribes nigrum L.). Microbiol Immunol 56: 805–809. 22985050 10.1111/j.1348-0421.2012.00510.x

[16] Gopalan A, Reuben SC, Ahmed S, Darvesh AS, Hohmann J, et al (2012) The health benefits of blackcurrants. Food Funct 3: 795–809. 22673662 10.1039/c2fo30058c

[17] Garbacki N, Tits M, Angenot L, Damas J (2004) Inhibitory effects of proanthocyanidins from Ribes nigrum leaves on carrageenin acute inflammatory reactions induced in rats. BMC Pharmacol 4: 25. 15498105 10.1186/1471-2210-4-25 PMC526370

[18] Tabart J, Kevers C, Evers D, Dommes J (2011) Ascorbic acid, phenolic acid, flavonoid, and carotenoid profiles of selected extracts from Ribes nigrum. J Agric Food Chem 59: 4763–4770. 21417457 10.1021/jf104445c

[19] Tits M, Poukens P, Angenot L, Dierckxsens Y (1992) Thin-layer chromatographic analysis of proanthocyanidins from Ribes nigrum leaves. J Pharm Biomed Anal 10: 1097–1100. 1298369 10.1016/0731-7085(91)80128-v

[20] Seyer R, Hrincius ER, Ritzel D, Abt M, Mellmann A, et al (2012) Synergistic adaptive mutations in the hemagglutinin and polymerase acidic protein lead to increased virulence of pandemic 2009 H1N1 influenza A virus in mice. J Infect Dis 205: 262–271. 22102733 10.1093/infdis/jir716

[21] Ehrhardt C, Kardinal C, Wurzer WJ, Wolff T, von Eichel-Streiber C, et al (2004) Rac1 and PAK1 are upstream of IKK-epsilon and TBK-1 in the viral activation of interferon regulatory factor-3. FEBS Lett 567: 230–238. 15178328 10.1016/j.febslet.2004.04.069

[22] Basler CF, Wang X, Muhlberger E, Volchkov V, Paragas J, et al (2000) The Ebola virus VP35 protein functions as a type I IFN antagonist. Proc Natl Acad Sci U S A 97: 12289–12294. 11027311 10.1073/pnas.220398297 PMC17334

[23] Nicholls JM, Chan RW, Russell RJ, Air GM, Peiris JS (2008) Evolving complexities of influenza virus and its receptors. Trends Microbiol 16: 149–157. 18375125 10.1016/j.tim.2008.01.008

[24] Eierhoff T, Hrincius ER, Rescher U, Ludwig S, Ehrhardt C (2010) The epidermal growth factor receptor (EGFR) promotes uptake of influenza A viruses (IAV) into host cells. PLoS Pathog 6: e1001099. 20844577 10.1371/journal.ppat.1001099 PMC2936548

[25] Eierhoff T, Ludwig S, Ehrhardt C (2009) The influenza A virus matrix protein as a marker to monitor initial virus internalisation. Biol Chem 390: 509–515. 19335204 10.1515/BC.2009.053

[26] Feld M, Shpacovitch VM, Ehrhardt C, Kerkhoff C, Hollenberg MD, et al (2008) Agonists of proteinase-activated receptor-2 enhance IFN-gamma-inducible effects on human monocytes: role in influenza A infection. J Immunol 180: 6903–6910. 18453611 10.4049/jimmunol.180.10.6903

[27] Khoufache K, Berri F, Nacken W, Vogel AB, Delenne M, et al (2012) PAR1 contributes to influenza A virus pathogenicity in mice. J Clin Invest. 123: 206–214. 10.1172/JCI61667 PMC3533265 23202729

[28] Khoufache K, LeBouder F, Morello E, Laurent F, Riffault S, et al (2009) Protective role for protease-activated receptor-2 against influenza virus pathogenesis via an IFN-gamma-dependent pathway. J Immunol 182: 7795–7802. 19494303 10.4049/jimmunol.0803743

